# Peripheral Neuropathy as a Complication of SARS-Cov-2

**DOI:** 10.7759/cureus.11452

**Published:** 2020-11-12

**Authors:** Britta L Bureau, Ahmed Obeidat, Mohan S Dhariwal, Pinky Jha

**Affiliations:** 1 Internal Medicine/Neurology, Medical College of Wisconsin, Wauwatosa, USA; 2 Neurology, Medical College of Wisconsin, Wauwatosa, USA; 3 Internal Medicine, Medical College of Wisconsin, Wauwatosa, USA

**Keywords:** covid-19, sars-cov-2, peripheral neuropathy, neurological symptoms, pain, post-viral

## Abstract

Previous reports have shown various neurological manifestations in about 36.4% of patients infected with SARS-Cov-2. However, peripheral neuropathy was only reported once before.

A 40-year-old healthy woman presented with two weeks of cough, nasal congestion, sore throat, intermittent fevers, fatigue, and myalgia but no weakness. She tested positive for the SARS-Cov-2. Physical exam showed no neurologic deficit. Two weeks later, respiratory symptoms were improving but she developed sudden leg pain, numbness, and weakness. She described it as a “pain crisis”. Neurological exam showed bilateral symmetrical, non-ascending lower extremity weakness and normal, symmetric reflexes. She had normal magnetic resonance imaging of the brain and spine, spinal fluid analysis, serum studies including creatinine kinase and C-reactive protein. She had elevated lactate dehydrogenase, low serum copper (72.9 (ref: 80.0-155.0 ug/dL)) and low vitamin B6 (14.6 (ref: 20.0-125.0 nmol/L)). A diagnosis of SARS-Cov-2-associated peripheral neuropathy was considered. We pursued empiric treatment with intravenous steroids (1000 mg methylprednisolone for three days), followed by a total of 2 g/kg of intravenous immunoglobulins (IVIG) given over five days. Pain management was done with gabapentin and ketorolac. We replaced copper and vitamin B6. Six weeks later, she reported improvement and was closer to baseline, but she endorsed residual, exertional, mild bilateral lower extremity pain, numbness, and weakness.

Previous reports of treatment of SARS-Cov-2-associated neuropathy included corticosteroids and IVIG. Our patient saw the most symptomatic improvement with gabapentin. In our case, the preserved reflexes, lack of ascending pattern, sudden onset of symptoms, and normal cerebrospinal fluid (CSF) argued against Guillain-Barre syndrome. Copper deficiency can result in myelopathy but not peripheral neuropathy, so is unlikely the sole explanation. Awareness and early treatment of peripheral neuropathy in SARS-Cov-2 can result in improved clinical outcomes for patients.

## Introduction

The spectrum of neurologic complications following the novel coronavirus 2 (SARS-Cov-2) infection is ever expanding. COVID-19, caused by the infection with the SARS-Cov-2, has become a global pandemic affecting more than 210 countries and the number of confirmed and fatal cases is on the rise [1]. Neurological manifestations occur in about 36.4% of patients infected with SARS-Cov-2 and span several domains within the central and peripheral nervous system [2]. Subacute peripheral neuropathy has been rarely reported. To our knowledge, only one report exists in the literature.

## Case presentation

A healthy 40-year-old woman presented with two weeks of cough, nasal congestion, sore throat, intermittent fevers, myalgia, generalized weakness, and fatigue. She tested positive for SARS-Cov-2. Initial neurologic exam was normal. Two weeks later her pulmonary symptoms improved but she developed sudden, severe bilateral leg pain, numbness, and weakness. The pain originated in the lower back and hips with radicular features. Pain was burning, stabbing, and aching at times; it waxed and waned. At worst, she scored the pain at 10/10 and referred to as “pain crisis”. Her numbness and weakness affected her ability to ambulate. She had no relief despite the use of paracetamol, ibuprofen, heat/ice therapy, and oxycodone. Neurological exam showed bilateral, symmetrical, non-ascending lower extremity weakness with preserved and symmetric deep tendon reflexes. Her power was reported as symmetric 4/5 in most groups in lower extremities. While detailed sensory exam was lacking, the constellation of observations suggest that she had a finding of sensory ataxia. Her walking was out of proportion for the level of weakness. Magnetic resonance imaging (MRI) of the brain and total spine was unremarkable. Lumbar MRI was performed with and without contrast and did not show any nerve root enhancement. Spinal fluid studies were normal (including nucleated cell count and protein level). Electrocardiogram was normal. Serum studies included normal creatinine kinase and C-reactive protein, but mildly elevated serum lactate dehydrogenase (222 (ref: 135-214 U/L)), borderline low serum copper (72.9 (ref: 80.0-155.0 ug/dL)) and low vitamin B6 (14.6 (ref: 20.0-125.0 nmol/L). Electromyography (EMG) was not obtained.

We considered a diagnosis of SARS-Cov-2-associated peripheral neuropathy. EMG was planned to be done but because of her slow but continuous improvement it was not performed. We initiated empiric treatment with intravenous steroids at 1000 mg methylprednisolone for three days, followed by a total of 2 g/kg of intravenous immunoglobulins (IVIG) given over five days. We administered gabapentin at 100 mg nightly, duloxetine at 30 mg twice daily, tramadol at 50 mg, and as needed ketorolac resulting in some pain control. This was coupled with intensive rehabilitation and copper replacement. Steroids were prescribed in this case for pain and fatigue due to possible post-viral syndrome. After pulse therapy was given, a quick oral taper followed. Upon hospital discharge she was able to walk with bilateral assistance and did not need supplemental oxygen.

At week 4, her “pain crisis” resolved, but continued to have chronic pain rated at a 4-6/10. She had persistent myalgia, weakness, numbness, and tingling. We increased gabapentin to 200 mg nightly and continued duloxetine at 30 mg twice daily.

At week 6, she had improved neuropathic pain. We increased gabapentin to 400 mg nightly. Repeat SARS-Cov-2 test was negative. Neurological exam revealed normal strength and tone.

At week 8, she reported complete resolution of her pain and was able to walk unassisted. All medications were tapered.

## Discussion

We reported a case of mixed sensorimotor neuropathy in association with SARS-Cov-2 infection with near complete resolution with immune-modulation, symptomatic therapy and intensive rehab. As for objective findings, our patient had 4/5 strength (motor), exam with sensory ataxia (sensory), and subjective paresthesia and pain which made us conclude she had a mixed sensorimotor neuropathy. The spectrum of neurological manifestations in association with COVID-19 continues to expand. It remains unclear which factors are associated with increased risk of neurologic manifestation. It was suggested that neurological symptoms are more common in patients with severe disease [2] but not older age [3].

Transmission of SARS-Cov-2 occurs via contact with a symptomatic or asymptomatic person via close contact (droplets) or distant (aerosol particles), contaminated surfaces, or fecal transmission [1]. The exact mechanism of entry into the central nervous system (CNS) is unknown, but currently discussed routes include retrograde neuronal transport across infected neurons, entry via the olfactory nerve, infection of the vascular endothelium, or white blood cell migration across the blood-brain barrier [4, 5]. Although SARS-Cov-2 may have direct access to the CNS, only two cases have been reported with SARS-Cov-2 in cerebrospinal fluid [6]. One possible method of direct nervous system entry is via angiotensin-converting enzyme-2 (ACE2). SARS-Cov-2 attaches to this ACE2, which is most frequently found in the lungs but is also present on neurons and glial cells in the central nervous system [2]. Neuropathy could also be caused by the host’s immune response reacting to the viral infection. It may be possible for SARS-Cov-2 to cause a cytokine storm, characterized by the immune system’s response to SARS-Cov-2 by rapidly releasing cytokines [2]. This can result in respiratory failure and neurological symptoms. Zinc deficiency can lead to cytokine production as well as olfactory and gustatory impairments [2]. If the patient presents after the virus has been cleared from the respiratory system, it makes determining etiology even more difficult. Further studies will be needed to determine if SARS-Cov-2 neurological manifestations are a result of direct infection or as result of systemic inflammation in response to SARS-Cov-2 infection.

There is a wide range of clinical presentations in SARS-Cov-2, with differing degrees of severity from asymptomatic carriers to fatal cases. The most frequent symptoms of SARS-Cov-2 include fever, dry cough, sore throat, shortness of breath, myalgia, fatigue, chills, chest pain, diarrhea, nausea, and vomiting with the most common complications including pneumonia and acute respiratory distress syndrome [1]. Although respiratory symptoms are the characteristic finding of SARS-Cov-2, it can also cause neurological manifestations. Research reports from Wuhan, China showed that neurological manifestations were present in 36.4% of patients and more common in patients with severe disease (45.5%) [2]. According to another study from Pakistan, neurological symptoms are present in 18.9% of SARS-Cov-2 patients [3]. The neurological symptoms reported were headache (6%), vertigo (3.4%), numbness/paresthesia (3.1%), altered consciousness (2%), hyposmia/anosmia (1.4%), and encephalitis (0.9%). Rare neurological presentations include cerebrovascular events (0.6%), seizure (0.3%), flaccid paralysis due to Guillain-Barré syndrome (GBS) (0.3%), and several case reports of Miller Fisher syndrome. These neurological manifestations typically occur in patients with a more severe course of SARS-Cov-2 [6]. Here we report a unique case of SARS-Cov-2 with peripheral neuropathy, which unlike prior documentation of SARS-Cov-2 paresthesia, neuropathy simultaneously includes motor and sensory involvement.

Our differential diagnosis included acute inflammatory demyelinating polyneuropathy (i.e., AIDP or Guillain-Barre syndrome), but it is possible that subacute peripheral neuropathy may be a manifestation of a post-viral inflammatory syndrome that is distinct from AIDP. Post-viral inflammatory syndrome has peak symptoms at onset followed by progressive improvement. Also, preserved deep tendon reflexes and the absence of a length dependence of symptoms argued against AIDP. Several case reports of SARS-Cov-2 develop Miller Fisher syndrome (MFS), a subtype of Guillain-Barré syndrome that can manifest days to a week after an upper respiratory tract infection. Our patient lacked the characteristic MFS triad of ataxia, ophthalmoplegia and areflexia [7]. One study suggested the potential of a post COVID-19 neuroimmune syndrome to include fatigue, diffuse myalgia, depressive symptoms, and disturbed sleep [8]. A diagnosis of vitamin B6 includes manifestations such as weakness, paresthesia, confusion, and sensory or dermatologic findings as well as physical exam findings such as stomatitis, glossitis, angular cheilitis, and seborrheic dermatitis [9]. Our patient did not have any of these findings. Symptoms of copper deficiency include leukopenia, bone and connective tissue abnormalities [10]; none of which our patient had. Copper deficiency can result in myelopathy but not peripheral neuropathy, so is unlikely the sole explanation. Her symptoms did not improve after vitamin B6 and copper supplementation, these were likely incidental findings due to poor PO intake after onset of SARS-Cov-2.

A case of peripheral neuropathy associated with SARS-Cov-2 was recently reported [11]. Like our case, this patient had mixed sensorimotor neuropathy, symptoms were worst on onset and progressively improved. He had distal lower extremity weakness and hyporeflexia including a 4/5 knee extension bilaterally, but normal motor in all other muscle groups. There was no sensory level, but there was gait ataxia [11]. This presentation differed in that he had no respiratory symptoms until seven days after onset of peripheral neuropathy. There was slow, but spontaneous (untreated) recovery of motor strength and gait [11]. Both this case and ours had maximal symptom at onset with slow, progressive improvement of symptoms making GBS unlikely. Our case differed in that it followed typical SARS-Cov-2 respiratory symptoms, making it likely a post-infectious immune-mediated neuropathy.

It is common knowledge that post-viral syndrome refers to a sense of fatigue one gets for weeks to months after fighting off a viral infection. One research study described the potential of a post COVID-19 syndrome following SARS-Cov-2 including symptoms such as chronic fatigue, diffuse myalgia, depressive symptoms, and disturbed sleep due to neuroimmune exhaustion [12]. Post-viral syndrome can take three courses: complete recovery, relapsing and remitting course due to stressors, or chronic symptoms. The most common cause of post-viral syndrome is Coxsackie virus, but other culprits include varicella, Epstein-Barr, and influenza virus due to viral mediated immune system damage [12]. A variant of post-viral syndrome called chronic fatigue syndrome/myalgic encephalomyelitis (CFS/ME) was documented following severe acute respiratory syndrome (another member of the coronavirus family), which prevented many people from returning to work for over 20 months [8]. Though not present in all, it is possible that peripheral neuropathy may be a symptom of post-viral syndrome. Early detection and symptomatic treatment in early stages of SARS-Cov-2 could help the patient overcome acute SARS-Cov-2 and prevent the onset of SARS-Cov-2 post-viral burden as well as prevent worsening the burden on healthcare facilities [8].

In 2002, SARS-Cov-1, similar to SARS-Cov-2 of 2019, emerged in China and spread throughout Asia [2]. In patients older than 65 years, mortality rate for SARS-Cov-1 was approximately 50%. There is limited published data, but neurological symptoms such as peripheral neuropathy and myopathy typically presented two to three weeks into the course of SARS-Cov-2 [2]. Similar to SARS-Cov-2, other symptoms included fever, chills, cough, diarrhea, and vomiting as well as olfactory and gustatory alteration dysfunction as initial symptoms of the disease [2]. As SARS-Cov-1 and 2 both use ACE2 to gain entry to host cells and have similar presentation of symptoms, it may be possible to predict neurological implications of SARS-Cov-2 using SARS-Cov-1 as a model.

Coronavirus is an enveloped, positive-sense, single stranded RNA virus that is part of the coronavirus family. A subspecies of the coronavirus family is called Middle East Respiratory Syndrome (MERS) which has also been implicated in neurological manifestations in rare cases. There have only been three documented cases of neuropathy in MERS, one of these cases presented similarly to our patient: 28-year-old orthopedic resident presented to the ED with four-day history of fever, generalized myalgia, dizziness and productive cough who tested positive for MERS-CoV. He had 13 days of fever and progressively declining respiratory function, due to weakness in both legs and inability to walk because of numbness and tingling in a stocking distribution [4]. Cerebrospinal fluid (CSF) analysis and MRI of head and whole spine were normal. Consultation with neurology diagnosed him with MERS-CoV-associated polyneuropathy and gave him IVIG 400 mg/kg daily for five days. When he was seen six months later he was continuing to make slow improvement [4]. SARS, another subspecies in the coronavirus family, also has documented cases of polyneuropathy [13].

Peripheral neuropathy can be relieved by opioids, but this can also worsen SARS-Cov-2 symptoms such as dry cough, fatigue, nausea, and gastrointestinal symptoms [14]. Additionally, any type of chronic pain (neck, back, orofacial, or headache) may necessitate an increased dose of analgesia. Prior research has shown that administration of ibuprofen can lead to decompensation and development of severe symptoms in early stage SARS-Cov-2 children, so ibuprofen should be avoided [14]. Of note, hydroxychloroquine often used for SARS-Cov-2 can cause adverse neurological side effects such as peripheral neuropathy, myopathy, irritability, and psychosis, so should be used cautiously if neurological symptoms may be present [5]. There are no clinical studies on the use of gabapentin for SARS-Cov-2-related neuropathic pain, but they are traditionally used in neuropathic pain, in general. While the number needed to treat (NNT) is similar for both, pregabalin is faster acting than gabapentin. Calcium channel blockers decrease respiratory drive, so they should be used carefully when prescribed in combination with opioids [14]. Other medications like duloxetine can be used solely or in combination. More research needs to be done, but possible neurological symptom improvement may come from zinc supplementation to prevent a cytokine storm. Since copper deficiency increases susceptibility to infections due to decreased number of critical immune cells, recent research has hypothesized that copper supplementation will boost the innate and adaptive immune systems in addition to its powerful antiviral properties [10]. Overall treatment should be an individualized approach based on the patient’s condition and pain level.

SARS-Cov-2 literature review was limited to articles published within 2020 to ensure current information. Limitations of this study include restriction of information from one patient and limited data collection due to the necessity of her self-isolation, the retrospective nature of the chart and literature review as well as the ever-changing knowledge base for SARS-Cov-2.

## Conclusions

Awareness, understanding, and early detection of peripheral neuropathy associated with SARS-Cov-2 can result in improved clinical outcomes for patients and the development of enhanced treatment. Although only a small percentage of SARS-Cov-2 patients develop peripheral neuropathy, in a large pandemic, this can have a large impact. Given the novelty of this viral infection, anecdotal experience in diagnosing and treating neurologic complications can add to the literature and help the practicing physician. A review of the current literature is included for a thorough analysis of peripheral neuropathy to guide clinical diagnosis and treatment. Further laboratory and clinical studies will be imperative to fully understand the pathogenesis and presentation of neurological symptoms. Long-term follow-up with careful assessment of the nervous system will be important to identify the long-term neurological ramifications of SARS-Cov-2.
